# Public Primary School Compliance with School Canteen Policy in Riyadh, Saudi Arabia: A Cross-Sectional Study

**DOI:** 10.3390/nu17050854

**Published:** 2025-02-28

**Authors:** Areej Alsiwat, George Kitsaras, Anne-Marie Glenny, Haya Alayadi, Michaela Goodwin

**Affiliations:** 1 Division of Dentistry, School of Medical Science, Biology, University of Manchester, Manchester M13 9PL, UK; georgios.kitsaras@manchester.ac.uk (G.K.); a.glenny@manchester.ac.uk (A.-M.G.); michaela.goodwin@manchester.ac.uk (M.G.); 2 Dental Health Department, College of Applied Medical Sciences, King Saud University, P.O. Box 10219, Riyadh 11433, Saudi Arabia; halayadi@ksu.edu.sa

**Keywords:** dental caries, cariogenic foods, school canteens, policy, school meals

## Abstract

**Background/Objectives:** The Ministry of Health and Education in Saudi Arabia established school canteen guidelines that have been mandatory since 2014. Only one previous study has investigated the compliance of public high schools with these guidelines. The aim of this study is to explore public primary school compliance with the school canteen policy guidelines set by the Saudi Ministry of Health and Education. **Methods:** A cross-sectional study with a total of 80 public primary schools randomly selected from five regions in Riyadh, Saudi Arabia, was conducted. School canteens were explored using a school canteen checklist provided by the Ministry of Education. The checklist contains a list of items grouped into healthy and unhealthy foods. A final assessment for alignment with the checklist was classified as either poor, average, or good. Canteen staff were provided with a structured questionnaire on the day of the school visit to gather their opinions and feedback regarding the canteen. **Results:** A total of 70% of schools showed an average rating for alignment with the Ministry of Health and Education guidelines; however, most of the items available in canteens were unhealthy. The canteen staff recommended adding healthier options like milk, fruit, and vegetables to school canteens. Furthermore, canteen staff reported issues in regard to expensive foods and a lack of choice in healthy food options. **Conclusions:** This study has shown that public primary schools in Riyadh, Kingdom of Saudi Arabia (KSA), have an average rating alignment with the Ministry of Health and Education’s school food policy guidelines. This study highlights the need for improvement in the Saudi Ministry of Education’s checklist, particularly regarding the availability of healthy foods, including milk, fruits, vegetables, and other nutritious items.

## 1. Introduction

During childhood and adolescence, nutrition is essential for optimal growth, health, and well-being throughout childhood and beyond [1]. The foundation for good nutrition is the early adoption of healthy eating habits, which have an impact on long-term health, including the prevention of non-communicable diseases (NCDs) in later life [1]. Unhealthy eating leads to weight gain and non-communicable diseases (NCDs), such as diabetes, stroke, heart disease, and cancer [2,3], NCDs are responsible for 70% of deaths worldwide [4]. One of the most pressing issues affecting global public health today is childhood obesity. It is estimated that 38.3 million children under the age of 5 worldwide are overweight, with 36% living in low- and high-income countries [2]. In addition, an unhealthy diet and poor nutrition can affect oral health, leading to dental erosion and dental caries. These can cause pain and discomfort, affecting a child’s growth and well-being [5,6].

Good nutrition and a healthy diet are supported, promoted, and protected in large part by the school environment [7,8]. It has long been known that education and healthy eating go hand in hand. A child who eats well performs better in school, and a child who receives a good education builds a foundation for the future, which in turn helps countries develop economically and socially [9]. Nutrition policies in schools were generally regarded more favorably than other interventions for their ability to encourage healthy eating habits [10,11]. Numerous aspects, including the quality of food and drinks, the freshness of ingredients, flavor [12,13], menu diversity [14], and price fairness [15] influence students’ food choices and their satisfaction with the services provided by the canteen.

The Ministry of Health and Education in Saudi Arabia created “Regulations of Health Conditions for School Canteens” in 2004, which was revised in 2013; it has been mandatory for implementation since 2014. The regulations include a list of both approved and banned canteen food items [16]. Preference is given to items that adhere to the following nutritional criteria [17]: they must contain fewer than 200 calories per serving, with no more than 35% of total calories derived from fat (equivalent to a maximum of 70 kcal or 8 g of fat). Additionally, saturated fat must account for no more than 10% of total calories (or a maximum of 2.5 g), and the product must be free of hydrogenated and trans fats. Furthermore, total sugar content must not exceed 35% of total calories, corresponding to a maximum of 17.5 g per serving for a 200-calorie item. Lastly, sodium content must be less than 200 mg per serving. Banned canteen food items include sweetened beverages, chips, soda, sports drinks, all meat products, and fried foods [17].

To establish a baseline for the availability of cariogenic foods in primary school canteens, a cross-sectional study was carried out in Abha city, Saudi Arabia, in 2012 [18]. Eight public primary schools were selected, and all students within these schools were included in this study. A questionnaire was distributed to students, revealing that 83% of the food items available in school canteens were cariogenic. Additionally, 17% of children relied solely on food from the canteen, while 73% consumed food from both home and the canteen [18]. Furthermore, a 2019 study conducted in Riyadh, Saudi Arabia, assessed the compliance of 76 randomly selected public high schools for boys with the Saudi policy chapter on “Meals and Beverages Offered in School Canteens”. This study designed questionnaires for interviewing head teachers and canteen staff and included direct observation of the food offered in school canteens. Compliance was evaluated using a scoring system based on the presence or absence of banned items listed in the policy, along with adherence to the Institute of Medicine (IOM) standards. The findings revealed that compliance with the Saudi policy was only moderate, while alignment with IOM standards was low. This study highlighted the need for the Ministry of Education to enhance compliance with restrictions on other prohibited food items and to promote the availability of nutritious meals, such as whole grains, fruits, and vegetables [16].

The importance of school food policies in preventing non-communicable diseases cannot be overstated. The oral health and general health of students can be improved by schools bringing into practice evidence-based policies that encourage better food selections, limit the availability of sugary and unhealthy meals, and promote nutrition education. By providing priority to these programs, school food policies can lower the prevalence of obesity and dental caries, promote lifelong healthy eating habits, and ultimately enhance the general health of children. Even though Saudi Arabia has established school canteen guidelines that have been mandatory since 2014, only one study was carried out to investigate the compliance of public high schools with these guidelines [16]. The aim of this study is to explore public primary school compliance with the school canteen policy guidelines set by the Saudi Ministry of Health and Education in Riyadh, KSA. This aim will be achieved through the following objectives: (a) assess the food provided at schools against the school canteen checklist used by the Ministry of Health and Education, (b) identify the proportion of schools complying with school canteen guidelines, and (c) assess school canteen staffs’ opinion on the available food provided, including quality, cost, and choices. It is hypothesized that these schools have a good rating of compliance with the Saudi Ministry of Health and Education guidelines.

## 2. Materials and Methods

### 2.1. Study Design, Setting and Participants

This study is a cross-sectional study set within public primary schools in Riyadh, KSA, from September 2023 to November 2023. A list of all governmental public primary schools was obtained from the Ministry of Education. The sample size calculation was based on a number of 522 public primary schools in Riyadh, with 84% of schools reporting non-adoption of the Saudi food policy [16], a confidence level of 95%, and a margin error of +/−8%, requiring a minimum sample of 70 schools. A total of 80 schools were included in this study to account for any potential missing data. Schools were randomly selected from five different regions within Riyadh city (North, East, West, South, and Central) using stratified random sampling to ensure equal distribution of schools participating across the regions, and 16 schools were selected from each region.

### 2.2. Recruitment Process

This study received approval from the Ministry of Education to both obtain the lists of schools and access schools in Riyadh city. The principal investigator sent an invitation to the selected schools. Initial visits were conducted to formally present the project to the head teacher, providing a clear explanation of this study and its objectives. Unscheduled visits to explore the schools’ canteens were then carried out to reduce the possibility of any adjustments to the usual food provided at the canteen. A total of 20 dental hygienists were trained by the principal investigator on the protocol of data collection. Each dental hygienist was assigned to visit four schools.

### 2.3. Data Collection

Food policy in school canteens was scored according to its compliance with the Ministry of Health and Education guidelines. The Ministry of Education’s website provides the school cafeterias with a checklist that follows the Ministry of Health and Education guidelines (open access) [17]. The alignment scores (poor, average, good) were determined by the Ministry of Education, and the same scoring system was adopted for this study to ensure consistency with the Ministry of Education’s guidelines, which this study is comparing against [19]. A total of 28 items were included in the school cafeteria checklist (12 allowed healthy items, 16 not allowed unhealthy items), the school cafeteria received two points (+2) for each healthy item on the checklist seen in cafeterias and minus two (−2) for each non-healthy item available. The entire range will then be classified out of 56 points as either good (44–56 points), average (29–43 points), or poor (0–28 points). Furthermore, to identify differences based on availability in canteens, this study assessed healthy and non-healthy items from the checklist obtained individually. For healthy items, the entire range will then be classified out of 24 points as either good (19–24 points), average (13–18 points), or poor (0–12 points), and out of 32 points for non-healthy items as good (25–32 points), average (17–24 points), or poor (0–16 points).

School canteen staff opinions and feedback were assessed through a structured questionnaire. This questionnaire was developed, and once the preliminary version was completed, it was reviewed by an academic expert in the field. Then, to check for clarity and relevance, a pilot test was conducted with 10 canteen staff from 10 schools that were not included in the study sample. The objectives of the questionnaire were to assess the variety, quality, cost of items, and healthy choices in school cafeterias. Poor, fair, good, very good, and excellent were the ranges available for answering these questions. In addition, open questions were about items in the cafeteria they would like to see or remove and if they had further suggestions for improvement.

### 2.4. Data Analysis

SPSS version 29 (IBM) was used for data analysis. Descriptive statistics of categorical variables were presented as frequencies and percentages. Chi-squared (χ^2^) test was used to assess the relationship between the compliance of school canteens to the food policy guidelines and the regions where the schools are located.

### 2.5. Ethical Considerations

Data were encrypted and kept on servers or computers approved by the University of Manchester and backed up safely. All information related to this study was managed using unique ID numbers; during recruitment, ID numbers were issued, and they were used throughout this study. Anonymized data will be included in publications and conference presentations.

All necessary ethical approvals were in place. This study obtained approval from the University of Manchester Research Ethics Committee (UREC) (Ref. No. 2023-17126-29750) the Institutional Review Board at King Saud University (Ref. No. 23/0425/IRB), and the Ministry of Education in Saudi Arabia to both obtain a list of schools and access schools in Riyadh city (Ref. No. 4500028581).

## 3. Results

### 3.1. Schools’ Compliance with School Canteen Policy Guidelines Set by the Ministry of Health and Education

All 80 primary schools in Riyadh, KSA, that were randomly selected agreed to participate when contacted. The overall findings indicate that 70% (*n* = 56) of school canteens fall within the average rating of alignment, while 30% (*n* = 24) were rated as poor (Table 1).

#### Availability of Healthy Meals and Unhealthy Meals in School Canteens

Exploring the availability of healthy and unhealthy meals in school canteens, the findings revealed that 79 out of 80 schools (98.75%) were rated as poor in terms of healthy food availability (Table 2). Additionally, in terms of unhealthy meals, 60% (*n* = 48) of schools were considered good due to their ban on unhealthy food, while 40% (*n* = 32) fell within the average rate (Table 3).

Although unhealthy items were considered to be at a good level in terms of unavailability, a significant number of schools still offered certain unhealthy options. Specifically, 67 schools (83.75%) included fried popcorn, 54 schools (67.5%) provided various types of chocolate, both plain and chocolate-covered, and 29 schools (36.25%) offered sandwiches or pies containing custard, chocolate, toffee, or vanilla (Table 4).

### 3.2. The Relationship Between School Compliance and the Regions Where the Schools Are Located

This study found insufficient evidence of a relationship between school canteen compliance with school canteen guidelines and the regions where the schools are located, based on the Pearson Chi-squared test (*p* = 0.081).

### 3.3. School Canteen Staff Opinions and Feedback

Only 75 canteen staff agreed to participate in the survey (out of the 80 schools taking part) during the school visit, with each school having one canteen staff member.

#### 3.3.1. Variety of Food

For the variety of food available in the school canteen, canteen staff from 25 schools (33.33%) reported a good variety. Additionally, 20 canteen staff members (26.66%) rated the variety as excellent, while another 20 (26.66%) described it as very good.

#### 3.3.2. Quality of Food

The majority of canteen staff, 48% (*n* = 36), rated the quality of food provided in schools as excellent. Meanwhile, 28% (*n* = 21) reported the quality as very good, followed by 22.66% (*n* = 17) who described it as good.

#### 3.3.3. Cost of Food

A total of 32 canteen staff members (42.66%) reported that the cost of meals in the school canteen is excellent. In addition, 16 schools (21.33%) considered the cost to be very good, while only 3 schools (4%) felt that the costs were poor.

#### 3.3.4. Healthy Food Choices

Regarding the availability of healthy food, a total of 31 canteen staff members (41.33%) reported very good or excellent availability of healthy items in school canteens. Additionally, 36% reported good availability, while only eight staff members (10.66%) reported poor availability of healthy items in the canteens (Table 5).

#### 3.3.5. Canteen Staff Feedback

Nearly half of the canteen staff (49.33%) reported that most students purchase healthy food, while 38.66% noted that students tend to prefer unhealthy food. Additionally, 12.01% of staff observed no noticeable difference in student preferences. A total of 26.66% (*n* = 20) requested they would like to see milk in the canteen items, and 24% (*n* = 18) reported the need for fruits and vegetables for more healthy options in the school canteen. Only six schools answered yes to removing items in the cafeteria. The items identified for removal include chocolate, chips, biscuits, popcorn, and pizza. A couple of suggestions were reported by the staff canteen: providing equipment to prepare fresh food, lack of food variety due to the limited options provided by the Ministry of Health and Education checklist, lack of healthy food variety, high prices of items in the canteen, consider children’s preference and provide food companies from the Ministry of Education that understand the school canteen guidelines.

## 4. Discussion

This study adds to the emerging evidence regarding primary school canteen alignment with the Ministry of Health and Education guidelines, focusing on the city of Riyadh, Saudi Arabia. The result of this study indicates that 70% of school canteens received an average rating for their adherence to the Saudi school canteen guidelines, 98.75% of schools lacked healthy snack options, and none offered fruits or vegetables. These findings align with a 2019 study conducted in public high schools in Riyadh [16], which reported that 94.7% of schools demonstrated moderate compliance with Saudi school policies regarding the presence and absence of banned items, and 96.1% had low compliance with IOM guidelines. Additionally, this study also reported that none of the 76 public high schools offered fruits or vegetables in their canteens [16]. This contrasts with a 2012 study in Abha city, which analyzed student questionnaires from eight randomly selected primary public schools, revealing that 13% of schools offered fresh fruit in their canteens [18].

This study has also found that 60% of schools were assessed as “good” with regard to the lack of availability of non-healthy food according to the Ministry of Health and Education checklist. However, more than half of the schools did include some cariogenic items, and the lack of healthy items in the school canteens could mean that most of the items purchased by students are unhealthy. Furthermore, unscheduled visits to schools found some unhealthy items hidden in one of the school canteens included in this study. These findings are consistent with the research conducted in public high schools in Riyadh (2019) that found the majority of pre-packaged foods served for breakfast were energy-dense snacks such as cakes and muffins (98.7%), candies (96%), biscuits and cookies (93.4%), and chips (52.6%) [16]. Similarly, 87% of the food products offered in the Abha primary school canteens were high-energy goods such as candy, chips, cakes, cookies, pudding, and ice cream [18]. Furthermore, this study found that other unhealthy items that were not included in the checklist were available in canteens, such as hazelnut biscuits. This also supported the study carried out in Riyadh (2019) that found 31.5% of public high schools had carbonated, fortified, and flavored waters in their canteens, as they were not listed as banned items in the Saudi school policy checklist [16]. Lastly, our study found insufficient evidence of a relationship between school canteen compliance and the regions where the schools are located. This is consistent with the findings of previous studies [20,21].

The school canteen staff were asked for their opinions on the quality, variety, and cost of school meals, and they reported that meals were of good quality, offered a good variety, and had appropriate costs. However, this contrasts with previous studies that assessed students’ satisfaction with cafeteria quality. A 2019 study found that high school students had a moderate level of satisfaction with the canteen quality [22]. Additionally, a 2024 study showed that university students’ satisfaction with the quality, variety, and cost of meals was low [23]. In addition, the school canteen staff in this study reported that most of the meals were healthy, which contradicts the results of both this study and previous research [24,25]. This discrepancy may be due to social desirability bias and the way in which the data were collected.

Staff members in the canteens reported students’ preferences for both healthy and non-healthy food similarly across all schools. Only six school canteen staff asked for unhealthy items like chocolate, chips, and biscuits to be removed, and 38 staff members recommended adding healthier options like milk, fruit, and vegetables. Furthermore, the staff brought up the issues of expensive foods and a lack of choice in healthy food options. These suggestions could be addressed through contracting with companies that follow the school canteen guidelines [26]. In addition, students’ preferences should also be taken into account, as they are the first consumers, and their view of the school canteen is important [27,28,29]. This is similar to what has been suggested in the checklist for headteachers provided in the UK [30]. They emphasized how crucial it is to sign the proper contracts with companies that follow the rules of the school canteen. Additionally, they have emphasized how crucial it is for teachers and students to communicate effectively in order to learn about each other’s perspectives on school cafeterias [30].

### 4.1. Strength

It is believed that this study is the first of its type to look at how well Saudi Arabia’s policy controlling “Meals and beverages offered in school canteens” is being followed by public primary schools in Riyadh, KSA. In addition to the large number of schools from different regions, it included unscheduled visits, which restricted the ability of schools to remove or replace any food items during the school visit.

### 4.2. Limitation

Since our study was limited to Riyadh, its findings cannot be generalized to schools in other Saudi Arabian cities. Second, as our study only looked at public primary schools, we are unable to generalize the findings to Riyadh’s public middle, elementary, and private schools. A further limitation is the lack of existing comparable research on this topic, particularly within the study region. Furthermore, some staff members may have been unwilling to speak negatively about the school, even with assurances of anonymity and confidentiality, which could have affected their responses.

### 4.3. Recommendation

More research is required on how best to ensure canteen guidelines are implemented effectively. Further studies to evaluate private schools’ alignment with the Ministry of Health and Education school canteen guidelines are needed. Studies on the regulations that schools have regarding home-brought meals also need to be evaluated. Parents and teachers have a significant influence on a child’s behavior and choices; more studies should be carried out to assess their nutritional knowledge, attitudes, and practices. Furthermore, it is also recommended to evaluate the opinions of schoolchildren, as their perspectives are valuable in assessing school canteen services.

## 5. Conclusions

This study has shown that public primary schools in Riyadh, KSA, have an average rating compliance with the Ministry of Health and Education’s school food policy guidelines. This study highlights the need for improvement in the Saudi Ministry of Education’s checklist, particularly regarding the availability of healthy foods, including milk, fruits, vegetables, and other nutritious items. Furthermore, this study supports education and health stakeholders, as its findings can help ensure effective implementation of nutrition policies, advocate for stronger policies, and promote student well-being.

## Figures and Tables

**Table 1:** School compliance with school canteen policy guidelines.

School Category According to Their Alignment	*n*
Poor	24 schools (30%)
Average	56 schools (70%)
Good	No schools

**Table 2:** Availability of healthy meals in school canteens.

Availability of Healthy Meals in School Canteens	*n*
Poor	79 schools (98.75%)
Average	1 school (1.25%)
Good	No schools

**Table 3:** Ban on unhealthy meals in school canteens.

Ban on Unhealthy Meals in School Canteen	*n*
Poor	0 schools
Average	32 school (40%)
Good	48 schools (60%)

**Table 4:** Unhealthy items available in school canteens.

Unhealthy Items Included in School Canteens	*n*
Milk and yogurt with artificial flavors	3 schools (3.75%)
Falafel	16 schools (20%)
Sandwiches or pies containing custard, chocolate, toffee, or vanilla	29 schools (36.25%)
All kinds of chips and fried food with high heat	15 schools (18.75%)
Salted nuts	23 schools (28.75%)
Ice cream	3 schools (3.75%)
Fried Popcorn	67 schools (83.75%)
Sweets consisting of sugar (jelly, snack pal, lollipop, Choco bar)	14 schools (17.5%)
Puffs and blown corn fingers	9 schools (11.25%)
Chocolate of all kinds, plain and/or covered with chocolate	54 schools (67.5%)
Donuts of all kinds, plain and/or covered with chocolate	15 schools (18.75%)

**Table 5:** Canteen staffs’ opinions on the canteen service.

	Poor	Fair	Good	Very good	Excellent
Variety of food	2 (2.66%)	8 (10.66%)	25 (33.33%)	20 (26.66%)	20 (26.66%)
Quality of food	----	1 (1.33%)	17 (22.66%)	21 (28%)	36 (48%)
Cost of food	3 (4%)	7 (9.33%)	17 (22.66%)	16 (21.33%)	32 (42.66%)
Healthy choices	8 (10.66%)	9 (12%)	27 (36%)	10 (13.33%)	21(28%)

## Data Availability

The original contributions presented in this study are included in the article. Further inquiries can be directed to the corresponding authors.

## Abbreviations

The following abbreviations are used in this manuscript:

KSA	Kingdom of Saudi Arabia
NCD	non-communicable diseases
IOM	Institute of Medicine
